# Suppression of IL‐8‐Src signalling axis by 17β‐estradiol inhibits human mesenchymal stem cells‐mediated gastric cancer invasion

**DOI:** 10.1111/jcmm.12786

**Published:** 2016-03-06

**Authors:** Chung‐Jung Liu, Fu‐Chen Kuo, Chiu‐Lin Wang, Chao‐Hung Kuo, Sophie S.W. Wang, Chiao‐Yun Chen, Yaw‐Bin Huang, Kuang‐Hung Cheng, Kazunari K. Yokoyama, Chun‐Lin Chen, Chien‐Yu Lu, Deng‐Chyang Wu

**Affiliations:** ^1^ Division of GastroenterologyDepartment of Internal MedicineKaohsiung Medical University HospitalKaohsiungTaiwan; ^2^ Center for Stem Cell ResearchKaohsiung Medical UniversityKaohsiungTaiwan; ^3^ School of MedicineCollege of MedicineI‐Shou UniversityKaohsiungTaiwan; ^4^ Department of Obstetrics and GynecologyE‐Da HospitalKaohsiungTaiwan; ^5^ Department of Obstetrics and GynecologyKaohsiung Municipal Hsiao‐Kang HospitalKaohsiungTaiwan; ^6^ Department of MedicineCollege of MedicineKaohsiung Medical UniversityKaohsiungTaiwan; ^7^ Department of Medical ImagingKaohsiung Medical University HospitalKaohsiungTaiwan; ^8^ Department of RadiologyCollege of MedicineKaohsiung Medical UniversityKaohsiungTaiwan; ^9^ Graduate Institute of Clinical PharmacyCollege of PharmacyKaohsiung Medical UniversityKaohsiungTaiwan; ^10^ Institute of Biomedical SciencesNational Sun Yat‐Sen UniversityKaohsiungTaiwan; ^11^ Graduate Institute of MedicineKaohsiung Medical UniversityKaohsiungTaiwan; ^12^ Department of Biological ScienceNational Sun Yat‐sen UniversityKaohsiungTaiwan; ^13^ Department of Internal MedicineKaohsiung Municipal Ta‐Tung HospitalKaohsiungTaiwan

**Keywords:** 17β‐estradiol, mesenchymal stem cell, IL‐8, Src, gastric cancer, cell invasion motility

## Abstract

Epidemiologic data show the incidence of gastric cancer in men is twofold higher than in women worldwide. Oestrogen is reported to have the capacity against gastric cancer development. Endogenous oestrogen reduces gastric cancer incidence in women. Cancer patients treated with oestrogens have a lower subsequent risk of gastric cancer. Accumulating studies report that bone marrow mesenchymal stem cells (BMMSCs) might contribute to the progression of gastric cancer through paracrine effect of soluble factors. Here, we further explore the effect of oestrogen on BMMSCs‐mediated human gastric cancer invasive motility. We founded that HBMMSCs notably secrete interleukin‐8 (IL‐8) protein. Administration of IL‐8 specific neutralizing antibody significantly inhibits HBMMSCs‐mediated gastric cancer motility. Treatment of recombinant IL‐8 soluble protein confirmed the role of IL‐8 in mediating HBMMSCs‐up‐regulated cell motility. IL‐8 up‐regulates motility activity through Src signalling pathway in human gastric cancer. We further observed that 17β ‐estradiol inhibit HBMMSCS‐induced cell motility *via* suppressing activation of IL8‐Src signalling in human gastric cancer cells. 17β‐estradiol inhibits IL8‐up‐regulated Src downstream target proteins including p‐Cas, p‐paxillin, p‐ERK1/2, p‐JNK1/2, MMP9, tPA and uPA. These results suggest that 17β‐estradiol significantly inhibits HBMMSCS‐induced invasive motility through suppressing IL8‐Src signalling axis in human gastric cancer cells.

## Introduction

Mesenchymal stem cells (MSCs) as an important population of cells that contribute to tumour progression and drug resistance 1, 2. Mesenchymal stem cells can migrate to the damaged tissue and the microenvironment of developing gastric tumour 3, in which MSCs might promote tumour malignance through tumour growth, invasion, metastasis formation and therapy resistance, through paracrine secretion of cytokines, chemokines, growth factors and the differentiation of MSCs into tumour‐associated fibroblasts 1, 4, 5, 6. So far, gastric carcinoma is one of the most prevalent cancers worldwide and is the secondary leading cause of cancer‐related mortality 7 because of the poor prognosis of patients. Invasion and metastasis are the major causes of death from human gastric cancer.

Interleukin‐8 (IL‐8 or called CXCL8), a cytokine of the CXC chemokine family, has been proposed to contribute to chronic inflammation and cancer development. Human mesenchymal stem cells (hMSCs) enhance cell motility of human HLaC78 head and neck squamous cell carcinoma cell line and IL‐8 cytokine secretion 8. High expression of IL‐8 in adipose‐derived stem cells (ASCs) may support breast tumour growth and progression 9. Interleukin‐8 may play a critical role in regulating the progressive growth of human gastric carcinoma cells 10. Overexpression of IL‐8 promotes the capacity of cellular adhesion, migration, invasion and chemoresistance in the gastric cancer cells 11. These findings suggest that IL‐8 might be a therapeutic target for gastrointestinal cancers.

Src family kinases play key roles in the development and progression of gastric cancer due of the elevated Src kinase activity in human gastric carcinoma tissues 12. Src‐dependent pathway regulates chemotaxis in response to a IL‐8 gradient in neutrophils 13, 14. Src family kinases can affect cell adhesion and migration through regulating activation of Cas and paxillin scaffold proteins 15. Studies reported that deregulation of Cas signalling is involved in stomach cancer progression and developmental defects in human 16. Paxillin that is demonstrated as a key regulator of the cellular migration machinery 2‐ and 3‐dimensional microenvironments has been recently regarded as the future prognostic and therapeutic target 17. Paxillin is activated and regulates normal neutrophil adhesion and motility when IL‐8 stimulation 18. Src/extracellularly responsive kinase (ERK) signalling is reported to be associated with gastric cancer progression 19, 20. Activation of c‐Jun N‐terminal kinase by N‐myc downstream‐regulated gene 1 (NDRG1) promotes tumour angiogenesis *via* VEGF‐A expression in gastric cancer 21. Thus, therapeutic strategies targeting Src hold promise for the treatment of gastric cancer.

Oestrogen against gastric cancer development has been reported such as that cancer patients treated with oestrogens have a lower subsequent risk of gastric cancer, and that the delayed menopause is associated with a reduced risk for gastric cancer development 22, 23. Hormone replacement therapy (HRT) has been reported protect against gastric cancer in women, even in men 24, 25. In the animal models of *N*‐methyl‐*N*′‐nitro‐nitrosogunidine‐induced gastric cancer, evidences also show a reduced incidence in female compared with male rats 26, 27. Oestrogen has been shown to impair CCL5‐ and IL6‐induced gastric cancer cell motility 28, 29, and reduce gastric cancer progression through suppression of erbB‐2 oncogene activation 24. These findings suggested that oestrogen might possess the protective effect against gastric cancer development. Oestrogen treatment is also reported to inhibit cell migration in human LoVo colon cancer cells 30, and attenuate hepatocellular carcinoma progression *in vitro* and *in vivo* 31.

In the present study, we investigated if 17β‐estradiol (E2) treatment is sufficient to inhibit human bone marrow mesenchymal stem cells (HBMMSCs)‐mediated invasive motility activity through impairing IL‐8 function, and further identified the related precise molecular and cellular mechanisms in human gastric cancer cells. The results demonstrated that 17β‐estradiol treatment inhibit HBMMSCS‐induced cellular motility by suppressing activation and expression of IL‐8‐up‐regulated Src downstream target proteins including phospho‐Cas, phospho‐paxillin, phospho‐ERK1/2, phospho‐JNK1/2, MMP9, tPA and uPA in human gastric cancer cells. This study suggests that 17β‐estradiol possesses the properties of anti‐cancer by inhibiting HBMMSCS‐mediated motility activity in human gastric cancer cells.

## Materials and methods

### Cells, antibodies, reagents and enzymes

Human gastric cancer cell lines (including AGS cells and CS12 cells) and HBMMSCs were obtained from the American Type Culture Collection (ATCC; Manassas, VA, USA) or from their originators. Human AGS gastric cancer cells were obtained from American Type Culture Collection/Bioresource Collection and Research Center (Taiwan) (ATCC^®^ Number: CRL‐1739^™^). Human bone marrow‐derived mesenchymal stem cells (ATCC^®^ PCS‐500‐012^™^) were obtained from the ATCC. Human CS12 gastric cancer cells were from the originator 32, 33, 34. 17β‐estradiol (E2) was purchased from Sigma (Sigma Chemical Co., St. Louis, Missouri, USA). The LY294002 (Akt inhibitor), U0126 (ERK1/2 inhibitor), SB203580 (p38 MAPK inhibitor), SP600125 (JNK1/2 inhibitor), XAV939 (beta‐catenin inhibitor), PP2 (Src inhibitor), PPT (ER‐alpha agonist) and DPN (ER‐beta agonist) were purchased from TOCRIS (Ellisville, MO, USA). Human cytokine array kit (Proteome Profile^™^ Array) was purchased from R&D Systems (Minneapolis, MN, USA). We utilized the following antibodies against phospho‐ERK1/2, ERK1/2, Src, phospho‐Src, phospho‐Cas, MMP9 (Cell Signaling Technology, Inc. Beverly, MA, USA); phospho‐JNK1/2, JNK1/2, Cas, phospho‐paxillin, paxillin, tPA, uPA, MMP2, ICAM‐1 and IL‐8RA/B (Santa Cruz Biotechnology, Inc. Santa Cruz, CA, USA); beta‐actin and alpha‐tubulin (Millipore, Billerica, MA, USA); ER‐alpha (Abcam, Cambridge, UK); ER‐beta (R&D Systems). Interleukin‐8 neutralizing antibody (R&D Systems) was used for specifically blocking human IL‐8 function. Goat isotype IgG was purchased from R&D Systems. Goat antimouse IgG antibody conjugated to horseradish peroxidase; goat anti‐rabbit IgG antibody conjugated to horseradish peroxidase and rabbit anti‐goat IgG horseradish peroxidase conjugate were purchased from Cell Signaling Technology, Inc.

### Cell culture

Human bone marrow mesenchymal stem cells were obtained from the American Tissue Culture Collection (ATCC^®^ Number: PCS‐500‐012^™^). Human bone marrow mesenchymal stem cells isolated from bone marrow were negative for CD13, CD34, CD45 and CD133, but were positive for CD29, CD44, CD73, CD90 and CD105. Human bone marrow mesenchymal stem cells were maintained and expanded in Iscove's modified Dulbecco's medium (IMDM; Gibco, Grand Island, NY, USA) and 10% foetal bovine serum (FBS; Hyclone, Logan, UT, USA) supplemented with 10 ng/ml basic fibroblast growth factor (FGF‐2; Sigma Chemical Co.), 5 ng/ml epidermal growth factor (EGF; R&D Systems), 100 units penicillin, 1000 units streptomycin and 2 mM L‐glutamine. Human bone marrow mesenchymal stem cells at passages 1–10 were used for the experiments in this study.

Human AGS cells (ATCC^®^ Number: CRL‐1739^™^) were cultured on 100‐mm or 60‐mm culture dishes in RPMI‐1640 medium supplemented with 100 μg/ml penicillin, 100 μg/ml streptomycin, 2 mM glutamine, 1 mM HEPS buffer and 10% FBS (all from Life Technologies, Carlsbad, CA, USA). Cell cultures were maintained at 37°C in a humidified 5% CO_2_ atmosphere. Adherent cells were detached from the culture dishes with trypsin/ethylenediaminetetraacetic acid (EDTA; Life Technologies).

Human CS12 cells from the originator 32, 33, 34 were maintained in humidified air (5% CO_2_) at 37°C and in the modified MCDB‐153 medium (kerotinocyte‐SFM; Gibco‐Invitrogen Corporation, Carlsbad, CA, USA) supplemented with *N*‐acetyl‐Lcysteine (NAC; 2 mmol/l) and L‐ascorbic acid 2‐phosphate (Asc 2P; 0.2 mmol/l), referred to as K‐NAC medium 32, 33, 34.

### Human cytokine array

Cell culture supernates form HBMMSCs were collected by centrifuging at 10,000 × g for 10 min. to remove cell debris. The membranes from human protein cytokine array kit (Proteome Profiler^™^ Array; R&D Systems) were blocked with blocking buffer at room temperature for one hour, and were followed by incubation with cell culture supernates overnight at 2–8°C on a rocking platform. The membranes were then washed with wash buffer, and incubated with the diluted Streptavidin‐HRP for 30 min. at room temperature. After washing, the membranes were assayed by chemiluminescence.

### Immunoblotting

To isolate total proteins, cells were washed with cold PBS and resuspended in lysis buffer (50 mM Tris, pH 7.5, 0.5 M NaCl, 1.0 mM EDTA, pH 7.5, 10% glycerol, 1 mM BME, 1% NP40) plus proteinase inhibitor cocktail and phosphatase inhibitor cocktail (Roche Molecular Biochemicals, Penzberg, Upper Bavaria, Germany). After incubation for 30 min. on ice, the supernatant was collected by centrifugation at 12,000 × g for 15 min. at 4°C, and the protein concentration was determined by the Bradford method. Sample containing equal proteins (40 μg) were loaded and analysed by immunoblotting. Briefly, proteins were separated by 12% SDS‐PAGE and transferred onto PVDF membrane (Life Technologies). Membrane were blocked with blocking buffer (5% non‐fat dry milk, 20 mM Tris‐HCl, pH 7.6, 150 mM NaCl and 0.1% Tween 20) for at least 1 hr at room temperature. Membranes were incubated with primary antibodies in the above solution on an orbit shaker at 4°C overnight. Following primary antibody incubations, membranes were incubated with horseradish peroxidase‐linked secondary antibodies (anti‐rabbit, antimouse or anti‐goat IgG). Antibody‐bound protein bands were detected using high sensitive Immobilon Western Chemiluminescent HRP Substrate (Millipore), and photographed with Bio‐Rad Chemiluminescence Imaging System (Bio‐Rad Laboratories, Inc. Hercules, CA, USA).

### Invasive motility assay

Cell invasive motility assay was performed with the 24‐well modified Boyden chambers containing membrane filter inserts with 8‐μm pores (Corning Incorporated Life Sciences, Tewksbury, MA, USA). Membrane filter inserts were pre‐coated with collagen I (Sigma Chemical Co.). To detect the effect of HBMMSCs on cell motility, the lower compartment was seeded with HBMMSCs, and gastric cells were placed in the upper chamber. The assay system was filled with serum‐ and phenol red‐free medium and incubated for 24 hrs in the presence of 17β‐estradiol, recombinant IL‐8 protein or IL‐8 neutralizing antibody. After incubation, invasive cells on the bottom of membrane filter were fixed with 4% paraforrmaldehyde and stained with 1 μg/ml 4′‐6‐diamidine‐2‐phenylindole dihydrochloride (DAPI; Roche Molecular Biochemicals) for 30 min. to detect cell nucleus (blue stains) by fluorescence microscopy. The motility phenotypes were determined by counting the cells that migrated to the lower side of the filter with fluorescence microscopy at 40× and 100× magnification respectively.

### ELISA

Conditioned medium (CM) from both of HBMMSCs culture and 17β‐estradiol‐treated HBMMSCs culture were collected by centrifuging at 10,000 × g for 10 min. to remove cell debris. The level of IL‐8 was measured using the ELISA kit obtained from R&D Systems according to the manufacturer's instructions.

### Cell proliferation assay

To determine the effect of 17β‐estradiol (E2) on gastric cancer cell viability, cells were treated with E2 (10^−10^, 10^−9^, 10^−8^ M) for 24, 48 and 72 hrs and were subjected to 3‐[4,5‐dimethylthiazol‐yl]‐2,5‐diphenyltetrazolium bromide (MTT; Sigma Chemical Co.) assay. The absorbance of blue formazan crystals was measured at 570 nm using an ELISA plate reader. The quantity of the formosan product was directly proportional to the number of viable cells in the culture medium. The cell viability of cells was determined according to the absorbance corrected to a background reading.

### Statistical analysis

Each experiment was repeated at least three times. Results were presented as the mean ± S.D., and statistical comparisons were made using the Student's *t*‐test. Significance was defined at the *P* < 0.05 or 0.01 levels.

## Results

### 17β‐estradiol suppresses HBMMSCs‐mediated cellular motility in human gastric cancer cells

**Figure 1:**
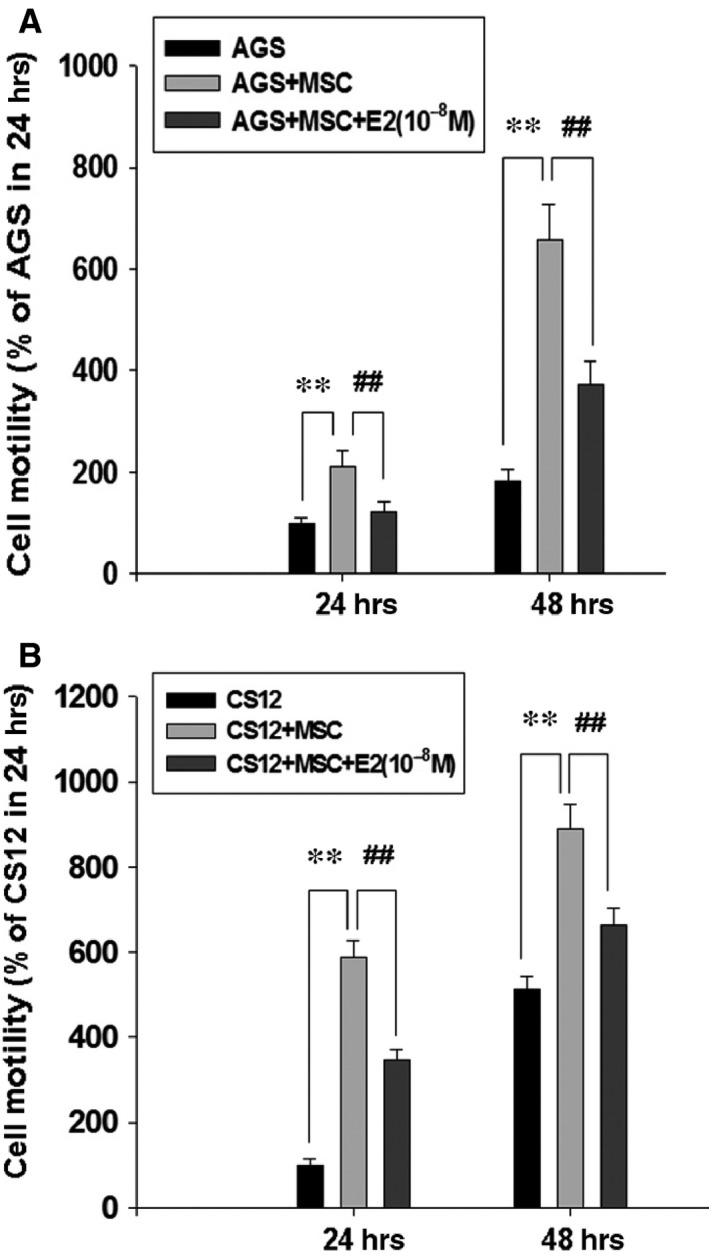
Inhibition of HBMMSCs‐induced cellular motility by 17β‐estradiol in human gastric cancer cells. Human bone marrow mesenchymal stem cells (HBMMSCs; 5 × 10^4^) and human gastric cancer cells (AGS, 5 × 10^4^ and CS12, 5 × 10^4^) were co‐culture with/without 17β‐estradiol (E2; 10^−8^ M) treatment for 24 and 48 hrs (**A** and **B**). The effect of 17β‐estradiol on HBMMSCs‐induced cellular motility in human gastric cancer cells was measured. **P < 0.01 *versus* control; ^##^ *P* < 0.01 *versus* only HBMMSCs co‐culture (mean ± S.D., *n* = 3).

The co‐culture system of HBMMSCs/gastric cancer cells was used to value the influence of 17β‐estradiol (E2) on HBMMSCs‐induced cellular motility in gastric cancer cells. In this study, we detected the effect of 17β‐estradiol (E2) on HBMMSCs‐increased motility activity in human gastric cancer cells by co‐culturing HBMMSCs and gastric cancer cells in the presence of E2 (10^−8^ M) for 24 and 48 hrs. Subsequently, we observed the ability of motility in gastric cancer cells by motility assay. In the motility assay (Fig. 1), the findings showed that E2 (10^−8^ M) notably inhibits HBMMSCs‐mediated motility activity in human AGS and CS12 cells.

### Analysis of secreted cytokines from HBMMSCs and human gastric cancer cells

**Figure 2:**
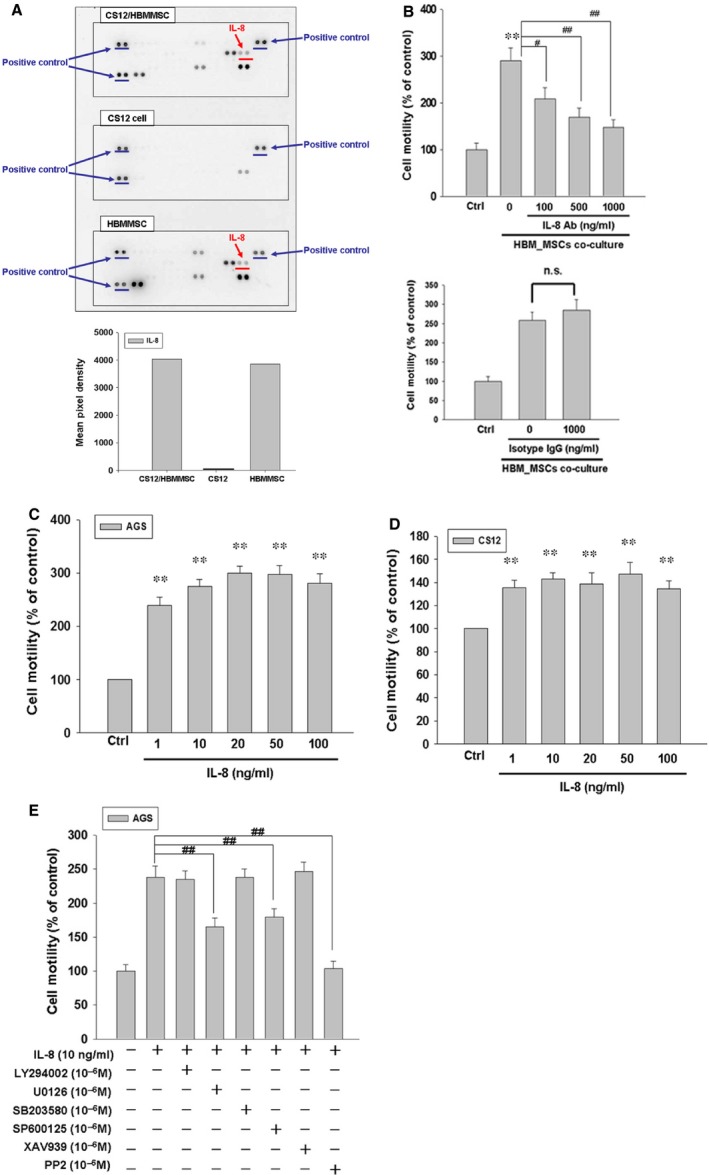
IL‐8 mediates HBMMSCs‐induced human cell motility *via* ERK, JNK and Src signalling pathways. HBMMSCs (5 × 10^4^) and human CS12 gastric cancer cells (5 × 10^4^) were cultured for human cytokine assay (**A**). HBMMSCs (5 × 10^4^) and human AGS cells (2.5 × 10^4^) were co‐culture with/without various IL‐8 neutralizing antibody. The effect of IL‐8 secreted from HBMMSCs on cellular motility of AGS gastric cancer cells was measured. The responses to different concentration of IL‐8 neutralizing antibody treatment were measured by the motility assay (**B**). Human gastric cancer cells (AGS and CS12) were treated with various concentration of recombinant IL‐8 (1, 10, 20, 50 and 100 ng/ml) for 16–24 hrs and subsequently measured the capacity of cell motility (**C** and **D**). AGS cells were pretreated with vehicle, LY294002 (Akt activation inhibitor, 1 μM), U0126 (ERK1/2 activation inhibitor, 1 μM), SB203580 (p38 MAPK inhibitor, 1 μM), SP600125 (JNK1/2 inhibitor, 1 μM), XAV939 (beta‐catenin inhibitor, 1 μM) or PP2 (Src inhibitor, 1 μM) for 1 hr and followed by recombinant IL‐8 (10 ng/ml) administration for 24 hrs. AGS cells were harvested and measured for cellular motility capacity (**E**). **P < 0.01 *versus* control (line 1); ^#^ *P* < 0.05; ^##^ *P* < 0.01 *versus* only HBMMSCs co‐culture or IL‐8 treatment (mean ± S.D., *n* = 3).

To determine which kind of cytokines were secreted by human (HBMMSCs) and gastric cancer cells in the culture medium, we used the human protein cytokine array to measure the cell culture supernates. Human bone marrow mesenchymal stem cells alone, CS12 cells alone and CS12 cells/HBMMSCs were, respectively, cultured for 24 hrs in serum‐ and phenol red‐free IMDM medium, samples of cell culture CM were collected for cytokine protein assay. The findings showed that HBMMSCs remarkably secreted IL‐8 soluble protein (Fig. 2A).

### IL‐8 neutralizing antibody inhibits HBMMSCs‐induced human AGS cell motility

In this study, we found IL‐8 was expressed from HBMMSCs in the highest level. To identify the effect of IL‐8 secreted from HBMMSCs on cellular motility activity in human gastric cancer cells, we used the specific neutralizing antibody to eliminate the function of IL‐8 cytokine. Co‐culture of HBMMSCs and AGS cells were established for valuing the effect of HBMMSCs on cellular motility in human gastric cancer cells. We found that HBMMSCs significantly contributed to cellular motility activity in AGS cells. However, the HBMMSCs‐increased motility activity in AGS cells was diminished when using various concentrations of IL‐8 neutralizing antibody in this co‐culture system (Fig. 2B). The findings suggested that IL‐8 secreted from HBMMSCs plays a critical role in the induction of cell motility in human gastric cancer cells.

### IL‐8 promotes motility activity in human gastric cancer cells

To further confirm the role of IL‐8 in increasing the motility activity in gastric cancer cells, we treated AGS and CS12 cells with various concentrations (0, 1, 10, 20, 50 and 100 ng/ml) of recombinant IL‐8 for 24 hrs. Subsequently, the motility activity of gastric cancer cells was valued by cellular motility assay (Fig. 2C and D). We observed that IL‐8 in various concentrations (1, 10, 20, 50 and 100 ng/ml) induce a significant increase in cell motility in human AGS and CS12 gastric cancer cells.

### ERK, JNK and Src signalling pathways mediate IL‐8‐induced AGS cell motility

To further identify which signal transduction pathway(s) was involved in the mechanism behind IL‐8‐up‐regulated motility activity in human gastric cancer cells, we applied the following inhibitors such as LY294002 (Akt inhibitor), U0126 (ERK1/2 inhibitor), SB203580 (p38 MAPK inhibitor), SP600125 (JNK1/2 inhibitor), XAV939 (β‐catenin inhibitor) and PP2 (Src inhibitor) to, respectively, block these pathways in the presence of IL‐8. AGS cells were pre‐incubated with LY294002 (10^−6^ M), U0126 (10^−6^ M), SB203580 (10^−6^ M), SP600125 (10^−6^ M), XAV939 (10^−6^ M) or PP2 (10^−6^ M) for 1 hr, followed by IL‐8 (10 ng/ml) treatment for 24 hrs and subsequently were subjected to motility assay. The finding showed that ERK1/2 inhibitor (U0126), JNK1/2 inhibitor (SP600125) and Src inhibitor (PP2) significantly suppress IL‐8‐induced motility activity of gastric cancer cells (Fig. 2E). The results suggested that ERK1/2, JNK1/2 and Src mediate IL‐8‐induced motility in human gastric cancer cells. We further found that PP2 possesses more inhibitory effect than U0126 and SP600125 in the motility assay.

### 17β‐estradiol inhibits IL‐8‐induced motility in human gastric cancer cells

**Figure 3:**
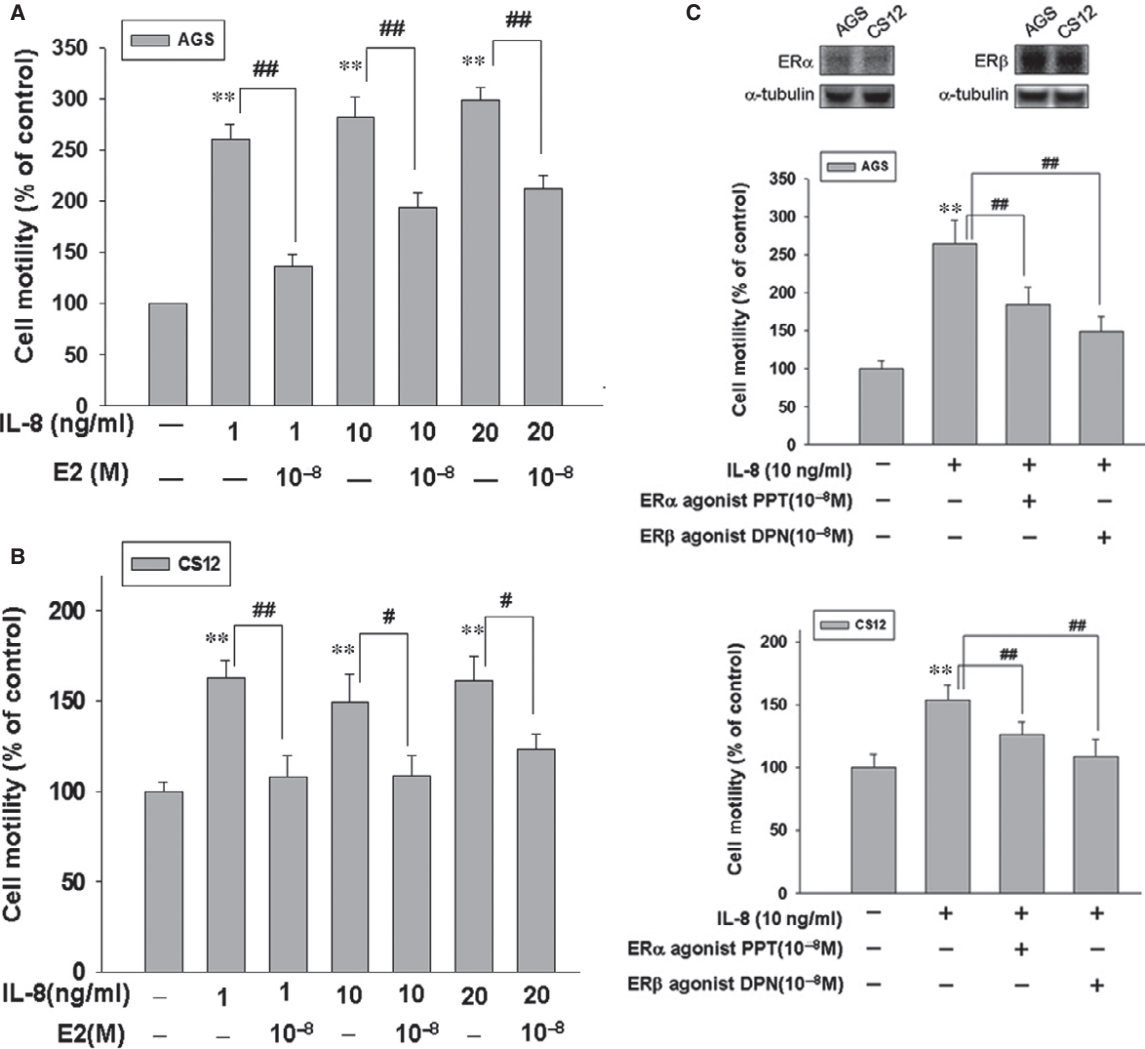
17β‐estradiol down‐regulates IL8‐induced motility activity in human AGS and CS12 gastric cancer cells. Human gastric cancer cells (AGS and CS12) were treated with various concentrations of IL‐8 (1, 10 and 20 ng/ml) in the absence or presence of 17β‐estradiol (10^−8^ M) for 16–24 hrs, then followed by measuring the capacity of cell motility. Agonists of ER‐α and ER‐β were also used for measure the inhibitory effect on invasive motility. *P* < 0.05; **P < 0.01 *versus* control (line 1); ^#^ *P* < 0.05; ^##^ *P* < 0.01 *versus* IL‐8 treatment (mean ± S.D., *n* = 3).

We explored the effect of 17β‐estradiol (E2) on IL‐8‐up‐regulated motility activity in human gastric cancer cells. We treated AGS and CS12 cells with various concentrations (0, 1, 10 and 20 ng/ml) of IL‐8 soluble protein in the presence of 17β‐estradiol (E2; 10^−8^ M) for 24 hrs. We observed that 17β‐estradiol (E2; 10^−8^ M) significantly inhibits IL‐8‐promoted motility activity in human AGS and CS12 gastric cancer cells (Fig. 3A and B). PPT (ER‐α agonist) and DPN (ER‐β agonist) were also used for measure the inhibitory effect on invasive motility. The results showed the significantly inhibitory effect of PPT and DPN on invasive cell motility in human gastric cancer cells (Fig. 3C).

### 17β‐estradiol inhibits IL‐8‐induced gastric cancer cell motility by suppressing activation of Src, Cas, Paxillin, ERK, JNK signalling pathways

**Figure 4:**
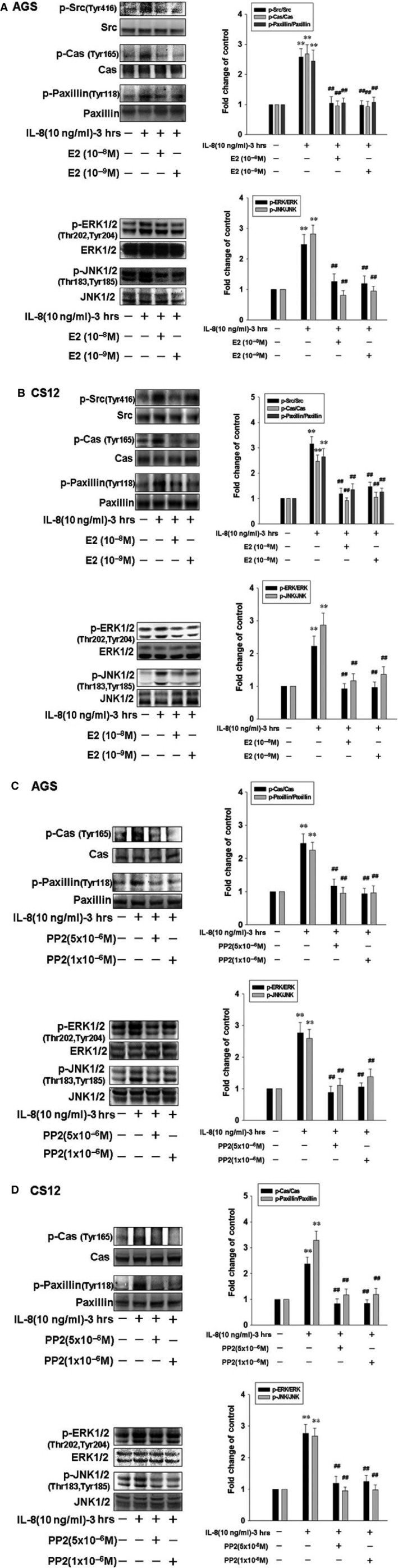
17β‐estradiol down‐regulates IL8‐induced motility activity by suppressing activation of Src, EKR and JNK signalling in human gastric cancer cells. Human gastric cancer cells were pre‐treated with 17β‐estradiol (10^−8^ and 10^−9^ M) or PP2 (Src activation inhibitor; 10^−6^ and 5 × 10^−6^ M) for 1 hr and followed by IL‐8 (10 ng/ml) treatment for 3 hrs. AGS and CS12 cells were then harvested for immunoblotting assay. **P < 0.01 *versus* control (line 1); ^##^ *P* < 0.01 *versus* IL‐8 treatment (mean ± S.D., *n* = 3).

We further treated gastric cancer cells with IL‐8 (10 ng/ml) in the presence of 17β‐estradiol (E2; 10^−8^ M) for 3 hrs, and measured the phosphorylation/activation of cancer progression‐related proteins by immunoblotting assay. Phosphorylation of Src, Cas, paxillin, ERK1/2 and JNK1/2 were significantly induced in response to IL‐8 stimulation. The activation status of Src, Cas, paxillin, ERK1/2 and JNK1/2 depends on the ratio of the phosphorylated and total proteins. The findings suggested that activation of Src, Cas, paxillin, ERK, JNK signalling pathways by IL‐8 might play an important role in mediating HBMMSCs‐induced gastric cell motility. However, IL‐8‐up‐regulated activation of Src (Tyr416), Cas (Tyr165), paxillin (Tyr118), ERK1/2 (Thr202, Tyr204) and JNK1/2 (Thr183,Tyr185) were significantly inhibited by 17β‐estradiol (E2; 10^−8^ and 10^−9^ M) treatment in human gastric cancer cells (Fig. 4A and B).

### Src mediates IL‐8‐induced activation of Cas, Paxillin, EKR and JNK signalling pathways in human gastric cancer cells

Here, we further identified the role of Src in IL‐8‐up‐regulated phosphorylation/activation of Cas, paxillin, ERK1/2 and JNK1/2 in human gastric cancer cells. AGS and CS12 cells were pre‐treated with PP2 (Src inhibitor; 10^−6^ M and 5 × 10^−6^ M) for 1 hr, and followed by the administration of IL‐8 (10 ng/ml) for 3 hrs, and subsequently were subjected to immunoblotting assay. We observed the elimination of phospho‐Cas (Tyr165), phospho‐paxillin (Tyr118), phospho‐ERK1/2 (Thr202, Tyr204) and phospho‐JNK1/2 (Thr183, Tyr185) after PP2 (10^−6^ M and 5 × 10^−6^ M) administration. Therefore, these data confirmed that Src mediates IL‐8‐induced cell motility and activation of Cas, Paxillin, EKR and JNK signalling pathways in human gastric cancer cells (Fig. 4C and D).

### 17β‐estradiol inhibits the expression of IL‐8‐Src signalling downstream invasive proteins MMP9, tPA and uPA in human gastric cancer cells

**Figure 5:**
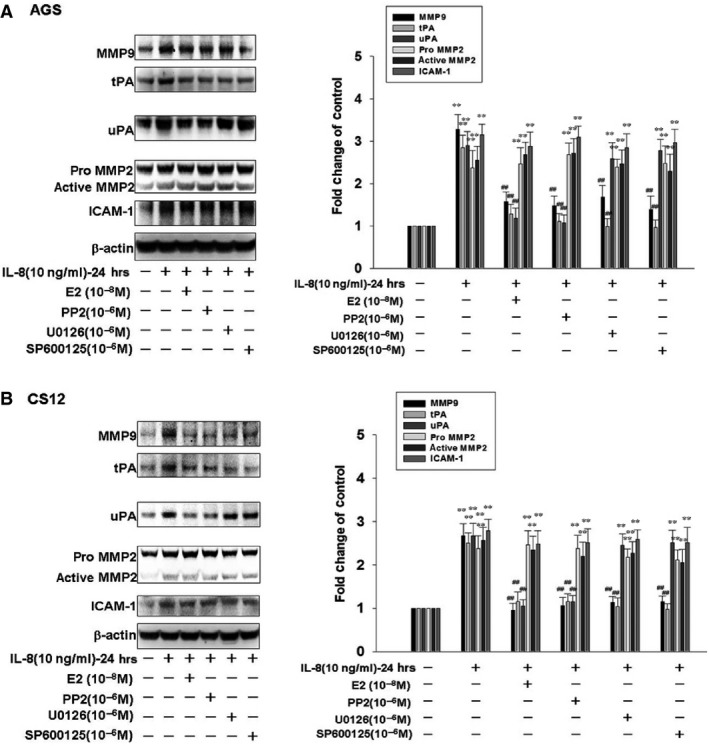
The affect of 17β‐estradiol on expression of IL8‐Src signalling downstream invasive proteins MMP9, tPA and uPA in human gastric cancer cells. Human gastric cancer cells were pre‐treated with 17β‐estradiol (10^−8^ M), PP2 (Src activation inhibitor; 10^−6^ M), U0126 (ERK1/2 activation inhibitor; 10^−6^ M) or SP600125 (JNK1/2 inhibitor; 10^−6^ M) for 1 hr and followed by IL‐8 (10 ng/ml) treatment for 24 hrs. AGS and CS12 cells were then harvested for immunoblotting assay. **P < 0.01 *versus* control (line 1); ^##^ *P* < 0.01 *versus* IL‐8 treatment (mean ± S.D., *n* = 3).

In this study, we investigated the influence of 17β‐estradiol on the invasive protein expression of IL‐8‐Src downstream signalling. Interleukin‐8 (10 ng/ml) treatment notably induced the expression of invasive proteins MMP2, MMP9, tPA, uPA and ICAM‐1. Interleukin‐8‐up‐regulated expression of MMP9 and tPA were suppressed by treatments of 17β‐estradiol (E2; 10^−8^ M), PP2 (10^−6^ M), U0126 (10^−6^ M) and SP600125 (10^−6^ M). Interleukin‐8‐induced expression of uPA were inhibited by treatments of 17β‐estradiol (E2; 10^−8^ M) and PP2 (10^−6^ M). These findings suggested that 17β‐estradiol inhibits cell motility through suppressing the expression of IL‐8‐Src signalling downstream invasive proteins MMP9, tPA and uPA in human gastric cancer cells (Fig. 5A and B).

## Discussion

**Figure 6:**
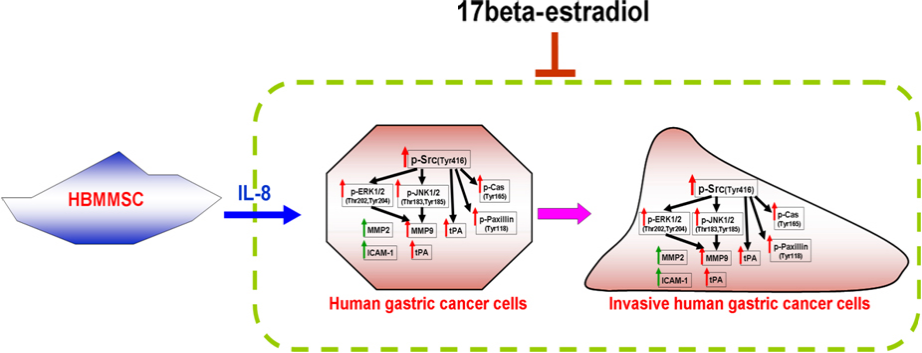
A schematic representation showing 17β‐estradiol inhibition of cell motility *via* suppression of IL8‐Src signalling axis in human gastric cancer cells. IL‐8 secreted from HBMMSCs induces the activation of Src, Cas and paxillin, contributing to the changes in organization of cellular actin cytoskeleton; up‐regulates ERK and JNK signalling pathways and the expression of invasive proteins MMP2, MMP9, tPA, uPA and ICAM‐1, which thus promotes invasive cell motility events in human gastric cancer cells. Src mainly mediates the activation of Cas, paxillin, ERK and JNK pathways and the high expression of invasive proteins MMP9, tPA and uPA in the presence of IL‐8. Treatment of 17β‐estradiol significantly inhibits HBMMSCs‐mediated invasive cell motility by suppressing activation of IL8**‐**Src signalling axis.

Major findings of this study can be summarized as followings: (*i*) Results from human cytokine arrays showed that HBMMSCs remarkably secrete IL‐8 soluble protein in cell culture CM. (*ii*) In the co‐culture system of HBMMSCs/AGS cells, treatment of IL‐8 specific neutralizing antibody inhibited HBMMSCs‐up‐regulated cell motility activity by blocking IL‐8 function. The role of IL‐8 in mediating cellular motility activity was further confirmed by using recombinant IL‐8 soluble protein. (*iii*) After administration of various specific inhibitors for cellular signalling pathways, we found that Src, ERK and JKN signalling pathways mediates IL‐8‐up‐regulated cell motility activity, and that Src mediates IL‐8‐induced activation of EKR and JNK signalling pathways in human gastric cancer cells. (*iv*) Co‐culture of gastric cancer cells/HBMMSCs in the presence of 17β‐estradiol (E2) showed 17β‐estradiol inhibition of HBMMSCs‐promoted motility activity in human gastric cancer cells. (*v*) Activation of Src by IL‐8 might play a critical role in mediating HBMMSCs‐up‐regulated motility activity. However, IL‐8‐up‐regulated cell motility activity and activation of Src, Cas, paxillin, ERK1/2 and JNK1/2 were significantly inhibited by17β‐estradiol (E2; 10^−8^ and 10^−9^ M). (*vi*) Furthermore, 17β‐estradiol inhibits the expression of IL‐8‐Src signalling downstream invasive proteins MMP9, tPA and uPA in human gastric cancer cells. These results demonstrated that 17β‐estradiol might inhibit HBMMSCs‐mediated human gastric cancer cell motility *via* suppression of IL‐8‐Src signalling axis (Fig. 6).

Mesenchymal stem cells recently have attracted attentions because of their capacity of migrating to and engrafting into the microenvironment of gastric tumour development. Studies have showed that MSCs can promote tumour growth by migrating to the developing intrahepatic cholangiocarcinoma through SDF‐1α/CXCR4 signalling pathway 35, and MMP2 molecular factor in human medulloblastoma 36, in which subsequently may lead to the effects of angiogenesis *via* VEGF, MCP‐1 and HIF‐1 signalling pathways. High expression of IL‐8 in ASCs may support breast tumour growth and progression 9. Increased IL‐8 in the bone microenvironment may represent one possible mechanism for microenvironment perversion in favour of acute lymphoblastic leukaemia cells 37. Interleukin‐8 has been proposed to contribute to chronic inflammation and cancer development. Interleukin‐8 may play an important role in regulating the progressive growth of human gastric carcinoma cells 10. Interleukin‐8 overexpression increases the capacity of cellular adhesion, migration, invasion and chemoresistance in the gastric cancer cells 11. *Helicobacter pylori*‐increased IL‐8 promotes human gastric cancer cell proliferation through transactivation of EGF receptor (EGFR) by disintegrin and metalloproteinase (ADAM) activation 38. In the present study, we observed HBMMSCs notably secrete IL‐8 protein in the human cytokine array. Human bone marrow mesenchymal stem cells‐up‐regulated motility activity of AGS cells was significantly inhibited by IL‐8 specific neutralizing antibody. Treatment of recombinant IL‐8 confirmed the influence of IL‐8 in mediating HBMMSCs‐up‐regulated cell motility. These findings suggested that IL‐8 secreted from HBMMSCs significantly increases cellular motility activity of human gastric cancer cells *via* the paracrine effect.

Gastric carcinogenesis is reported to be induced by active membrane‐bound receptors that lead to the activation of intracellular signalling pathways through multiple GTPase proteins, receptor kinases, kinases and phosphatases. The activation of phosphatidylinositide 3‐kinase (PI3‐K)/Akt (protein kinase B) and mitogen‐activated protein kinases (MAPKs) pathways occur during tumour development 39. Mitogen‐activated protein kinases include three major subfamilies such as the ERKs, the JNKs, also known as stress‐activated protein kinases, and the p38 MAPKs 39. Activation of β‐catenin signalling pathway is shown to mediate gastric cancer growth and invasion 40. Another pathway that has received attentions is Src pathway. Src plays a critical role in the variety of cancer cellular regulation such as cell division, motility, adhesion, angiogenesis and survival, so Src is regarded as the attractive target for future anti‐cancer therapeutics. Src kinase closely regulates the phosphorylation and activation of Cas adhesion protein and paxillin cytoskeletal protein, which operate cell migration in invasive cells during the development and pathological conditions associated cancer metastasis. Src‐dependent pathway has been show to regulate neutrophils chemotaxis in response to a IL‐8 gradient 13, 14. So targeting Src strategic therapies has been regarded as a great approach for gastric cancer. In this study, we observed that Src mediates IL‐8‐induced invasive motility in human gastric cancer cells. The critical role of Src was confirmed by using PP2, a specific inhibitor for Src, in the motility and immunoblotting assays. Compared with various signalling pathway inhibitors including LY294002 (PI3K/Akt activation inhibitor), U0126 (ERK1/2 activation inhibitor), SB203580 (p38 MAPK inhibitor), XAV939 (β‐catenin inhibitor) and SP600125 (JNK1/2 inhibitor), the findings indicated that Src mainly mediates IL‐8‐up‐regulated motility activity through modulating the activation of Cas, paxillin, EKR1/2 and JNK1/2 signalling pathways and the expression of invasive proteins MMP9, tPA and uPA in human gastric cancer cells.

Protective properties of oestrogen against gastric cancer development have been reported because of the reduced gastric cancer risk in the delayed menopausal women and in the HRT‐treated population compared with non‐treated one 22, 23. In the present study, we observed that 17β‐estradiol treatment significantly inhibited HBMMSCS‐mediated cell motility activity through suppressing IL‐8‐Src downstream target protein activation and expression, including Cas, paxillin, ERK1/2, JNK1/2, MMP9, tPA and uPA in human gastric cancer cells. 17β‐estradiol may inhibit HBMMSCS‐induced invasive motility through suppressing IL‐8‐Src signalling axis in human gastric cancer.

## Conclusion

Gastric cancer is one of the most common cancers worldwide, and is also the second leading cause of cancer‐related mortality. Accumulating evidence suggest that BMMSCs may contribute to the progression of cancer development through paracrine effect. Epidemiological data report that the incidence of gastric cancer in male is ~twofold higher than in women worldwide. Studies also report the delayed menopause is associated with a reduced risk for gastric cancer development. In this study, we further observed that 17β‐estradiol significantly inhibits HBMMSCS‐induced invasive motility through suppressing IL‐8‐Src signalling axis in human gastric cancer cells.

## Conflicts of interest

The authors declare that they have no competing interests.

## Supporting information


**Figure S1** 17β‐estrodial significantly inhibits human bone marrow mesenchymal stem cells (HBMMSCs)‐induced cell proliferation in human gastric cancer cells.
**Figure S2** The effects of 17β‐estrodial on the protein expression of IL‐8RΑ/Β in human gastric cancer cells.
**Figure S3** The effect of E2 alone on the human gastric cancer cells motility.
**Figure S4** In the present study, we detected the effect of 17β‐estradiol (E2) on HBMMSCs‐ increased motility activity in human gastric cancer cells by co‐culturing HBMMSCs and gastric cancer cells in the presence of E2 (10^−8^ M) for 24 hrs.
**Figure S5** The effect of 17β‐estrodial on IL‐8 secretion from HBMMSCs (using ELISA).
**Figure S6** Male NOD/SCID mice with 7 weeks of age were randomly assigned into 3 groups: AGS group (control), AGS+IL‐8 group, AGS+IL‐8 + E2 group.
