# Supplementary material for: Suppression of IL‐8‐Src signalling axis by 17β‐estradiol inhibits human mesenchymal stem cells‐mediated gastric cancer invasion
Source: J Cell Mol Med. 2016 Mar 6;20(5):962–72. doi: 10.1111/jcmm.12786 (PMC4831355; doi:10.1111/jcmm.12786)

**Supplementary Data 1**

17beta-estrodial significantly inhibits human bone marrow mesenchymal stem cells (HBMMSCs)-induced cell proliferation in human gastric cancer cells.


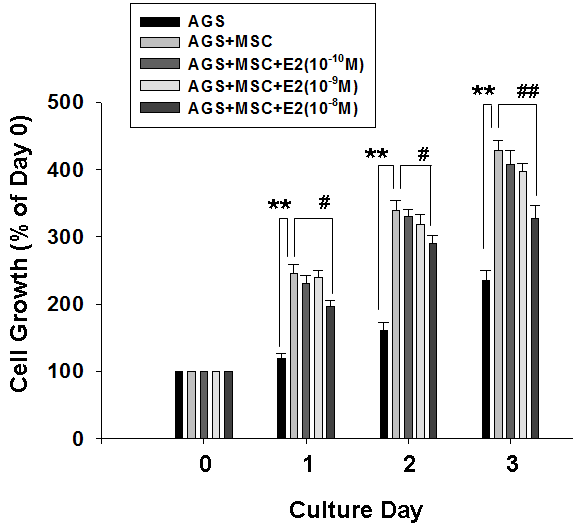


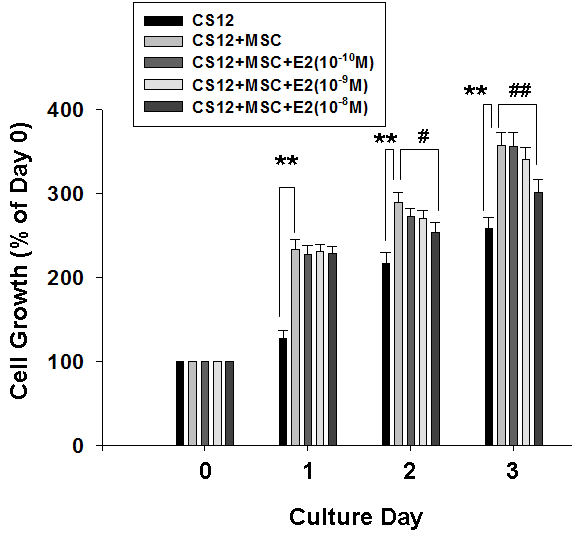


**Supplementary Data 2**

The effects of 17beta-estrodial on the protein expression of IL-8RA/B in human gastric cancer cells


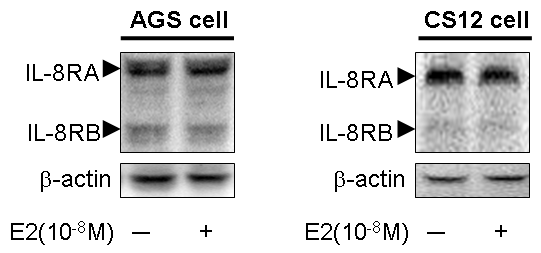


**Supplementary Data 3**

The effect of E2 alone on the human gastric cancer cells motility.


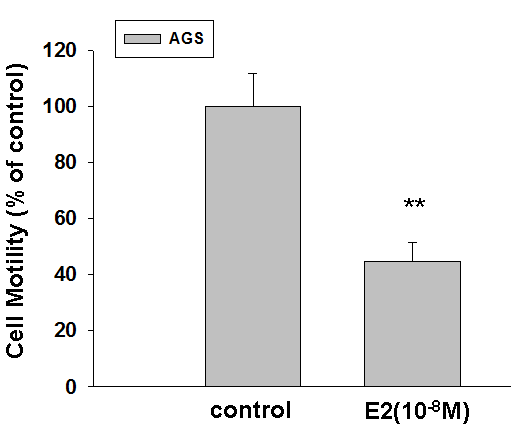


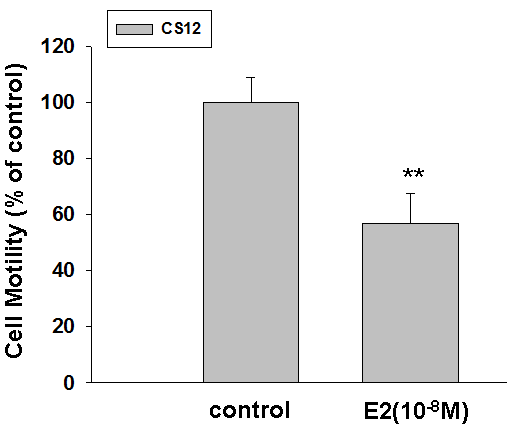


**Supplementary Data 4**

In the present study, we detected the effect of 17β-estradiol (E2) on HBMMSCs- increased motility activity in human gastric cancer cells by co-culturing HBMMSCs and gastric cancer cells in the presence of E2 (10-8M) for 24h. Gastric cancer cells themselves in the bottom as the control group (line1). In the motility assay, the findings showed that E2 (10^-8^M) notably inhibits HBMMSCs-mediated motility activity in human AGS and CS12 cells.


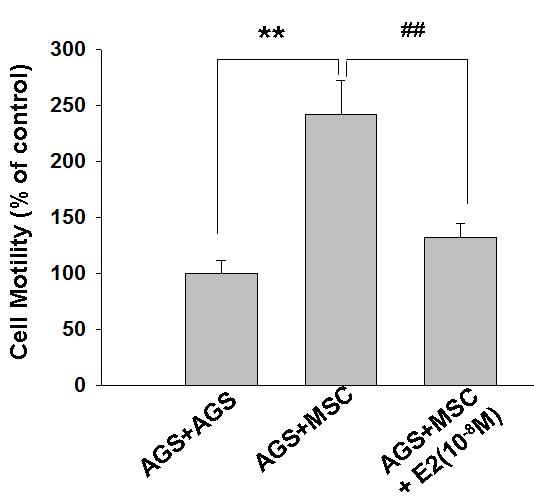


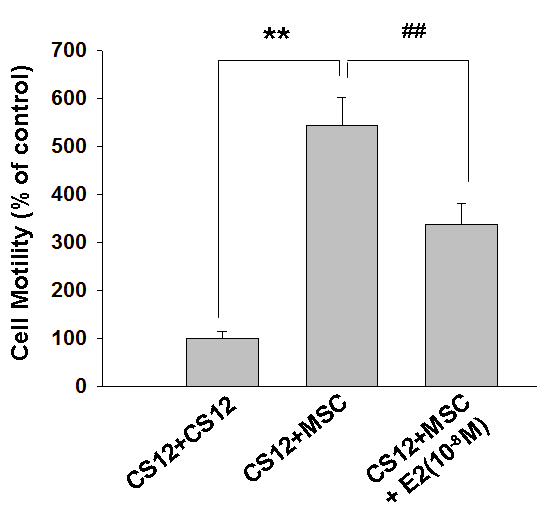


**Supplementary Data 5**

The effect of 17beta-estrodial on IL-8 secretion from HBMMSCs (using ELISA)


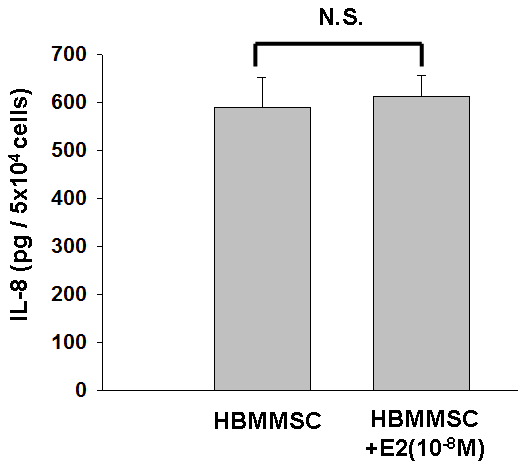


**Supplementary Data 6**

Male NOD/SCID mice with 7 weeks of age were randomly assigned into 3 groups: AGS group (control), AGS+IL-8 group, AGS+IL-8+E2 group. Tumors were established by the subcutaneous injection of 5×10^6^ AGS cells into the flanks of the mice. Tumor volume was estimated 2 times per week using width (*a*) and length (*b*) measurements (*a^2^b/2*, where *a* <*b*). 17beta-estradiol was solved in sesame oil. 17beta-estradiol (4ug/25g BW) and IL-8 (1ug/25g BW) were used for local multipoint injection.


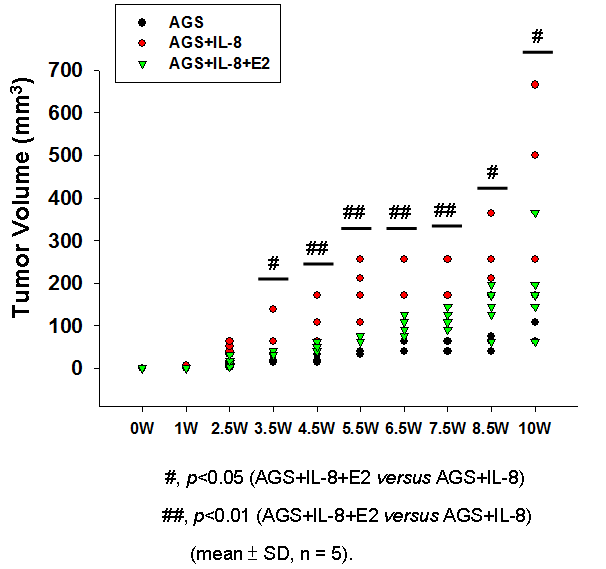


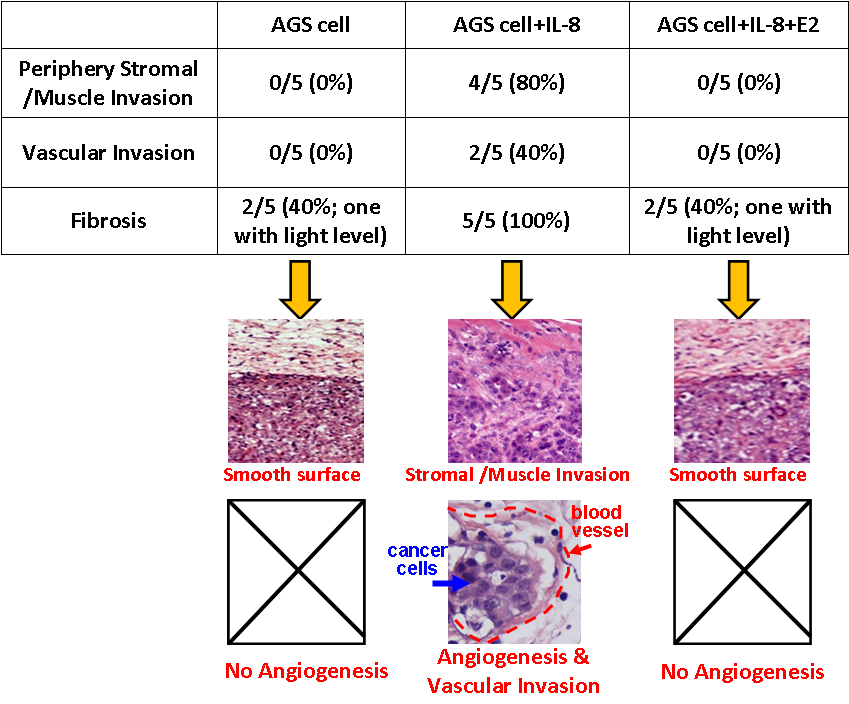

Supplement: Supplementary file 1 — Figure S1 17β‐estrodial significantly inhibits human bone marrow mesenchymal stem cells (HBMMSCs)‐induced cell proliferation in human gastric cancer cells. Figure S2 The effects of 17β‐estrodial on the protein expression of IL‐8RΑ/Β in human gastric cancer cells. Figure S3 The effect of E2 alone on the human gastric cancer cells motility. Figure S4 In the present study, we detected the effect of 17β‐estradiol (E2) on HBMMSCs‐ increased motility activity in human gastric cancer cells by co‐culturing HBMMSCs and gastric cancer cells in the presence of E2 (10−8 M) for 24 hrs. Figure S5 The effect of 17β‐estrodial on IL‐8 secretion from HBMMSCs (using ELISA). Figure S6 Male NOD/SCID mice with 7 weeks of age were randomly assigned into 3 groups: AGS group (control), AGS+IL‐8 group, AGS+IL‐8 + E2 group. [file JCMM-20-962-s001.doc]
